# Linguistic diversity in machine learning training data: Content analysis of English and Finnish soundscape descriptions in audio captioning

**DOI:** 10.1371/journal.pone.0350043

**Published:** 2026-06-11

**Authors:** Laura Hekanaho, Emilia Tuuri, Maija Surakka, Maija Hirvonen

**Affiliations:** 1 University of Helsinki, Languages, Helsinki, Finland; 2 Tampere University, Languages Unit, Tampere, Finland; University of Kurdistan Hewler, IRAQ

## Abstract

This article examines descriptions of auditory experiences used as training data in machine learning. While such training datasets play a key role in machine learning, only a few studies have investigated these training datasets from a linguistic perspective. Making an important contribution to the English-focused field, we compare descriptions produced in two typologically distant languages: English and Finnish. The English descriptions were produced by sighted participants, and the Finnish data both by sighted and non-sighted individuals, offering insight into variation in language use based on sensory experience. First, we provide an overview of the three datasets with a descriptive corpus analysis. The corpus analysis reveals greater variation among the Finnish corpora, in comparison with the English corpus. We attribute this to the use of L2 speakers as informants for the English data, considering potential data quality issues from a linguistic perspective. Second, we report on a qualitative content analysis conducted on a smaller sample of 150 descriptions, examining which elements the participants had identified and chosen to verbalize. In particular, the analysis indicates that the visually disabled participants make use of slightly richer lexical resources, especially in terms of verbalising spatial structure. In addition, a methodological finding of our study captures how the corpus-level and the discourse-level analyses lead to distinct, even seemingly contradictory results: while the corpus analysis focuses on token distributions, the qualitative discourse-level analysis reveals which different semantic domains are prevalent in different subcorpora. This article contributes to the growing research on cross-linguistic and language-internal diversity in the field of machine perception.

## Introduction

This paper examines human-generated descriptions of auditory experiences produced in English and Finnish. In recent years, the significance of language-based machine learning that seeks to imitate human intelligence (e.g., question-answering, [1]) has increased tremendously, especially with the commence of large language models such as the ChatGPT [2]. One key area of contemporary language-based machine learning is that of automatic captioning [3], with many relevant real-life applications. Many types of media, for example, can be made more accessible by automatic captioning [4].

Language-based machine learning is established by mapping statistical relationships in human-generated training data [5]. Thus, both the quantity and quality of the training data must be sufficient [3,6]. Yet, there are some well-known issues in obtaining quality training data [7], also in audio-based captioning [8]. To some extent, this may hinge on the fact that language users are more used to describing visual than auditory experiences, which also means that many languages are better equipped to describe the visual realm, reflected in a richer lexicon [9–11, p. 58].

As such, descriptions of auditory experiences provide a particularly interesting avenue for linguistic investigation, the results of which help better understand the nature of such descriptions and the challenges posed for machine learning. In particular, there is a call for more nuanced linguistic analyses that go beyond simple lexical analyses (see Discussion in [12,13]).

**Fig 1:**
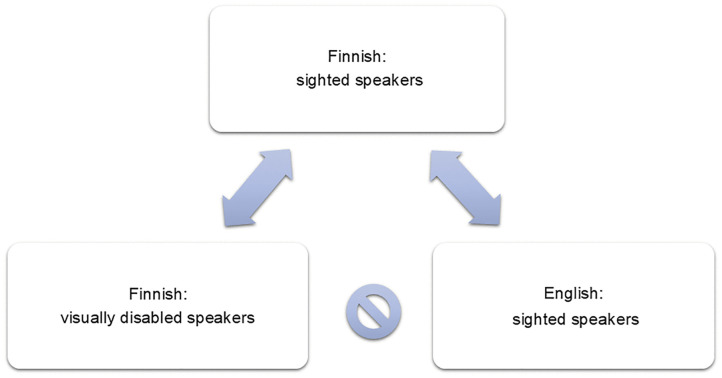
Comparative study design.

In this paper, we examine human-generated, written descriptions of audio clips, henceforth referred to as soundscape descriptions. With soundscapes we refer to a set of sounds that exist in a specific environment at a given time [13,14]. Following Kreiss et al. [15], we refer to our examples of language data as descriptions, understood as textual representations that could replace an audio clip (or image), instead of the often-employed catch-all “captions” used in machine learning. This study provides novel linguistic insight into soundscape descriptions, with two particularly important factors included in the study design: a comparison of Finnish and English descriptions, representing cross-linguistic diversity, and a comparison between sighted and visually disabled participants, representing language-internal diversity (Fig 1).

First, although there is a clear practical need for automatic captioning — and thus training data — in different languages, English has been dominant in automatic captioning studies [3]. Linguistically orientated investigations — which are altogether few — have also often focused on English training data [15,16]. Cross-linguistic endeavours seem to be even fewer, previous studies contrasting Germanic languages [17]. As such, this study makes an important contribution by comparing descriptions in two typologically distant languages: a Germanic language, English, and a Finno-Ugric language, Finnish. Second, especially since the description of auditory experiences seems to be more challenging than that of visual experiences, we examine soundscape descriptions produced by both sighted and visually disabled individuals. By necessity, visually disabled people are more adjusted to paying attention to auditory signals in their everyday lives (see Background), and as such, we expect visually disabled participants to produce richer soundscape descriptions.

Our research questions are: 1. What type of soundscape descriptions are produced by sighted and visually disabled individuals in Finnish, and by sighted participants in English? 2. What kind of linguistic diversity becomes observable via different methods, in this case corpus analysis and qualitative linguistic analysis? Concerning question 1, we focus primarily on the content of the descriptions, i.e., which information the participants have chosen to verbalize, paying some attention to the linguistic realizations as well. The content of the descriptions is an important factor from a machine learning perspective, since ideally, the participants will accurately identify and verbalize all relevant elements present in the audio clips [3]. Concerning question 2, although simple word frequency lists drawn from the training data will already show which elements have been identified by the participants in general, we demonstrate that a qualitative content analysis will produce more nuanced insights into the data. We proceed by discussing soundscapes and soundscape descriptions from a theoretical perspective in the Background section, alongside a short introduction of key differences between English and Finnish and a discussion of language use by visually disabled speakers. We then present our data and methods, before moving on to the analysis and final discussion of the results.

## Background

We begin by briefly characterizing the two languages under consideration, before turning our attention to soundscape studies and considering the role of visual disability in the act of describing soundscapes.

### Finnish and English

The Finnish language, belonging to the Finnic subgroup of the Uralic language family, is currently spoken by close to five million native speakers mainly in Finland. In contrast, English is considered a North Sea Coast language [18, pp. 17–18], traditionally classified as a West Germanic language belonging to the Indo-European language family [19]. Having had a lot of influence from Romance languages, English differs from more prototypical Germanic languages not only lexically but also grammatically, e.g., in terms of word order and lack of grammatical gender [18, p. 13].

Structurally, Finnish is an agglutinative language relying on affixes, mainly suffixes, in both verbal and nominal inflection [20]. Finnish uses a rich set of cases to code both core grammatical functions and adverbial relations [21]. The number of cases varies between 14–15 or even more in different accounts [20,22]: only about 15% of all languages employ more than 10 cases [23]. The agglutinative nature of Finnish is also manifested in its rich derivational morphology [20,24, pp. 353–357]. English, on the other hand, is considered an isolating, analytic language, making little use of any inflections [25], in great contrast to Finnish.

In terms of syntax, English belongs to SVO (Subject-Verb-Object), and although SVO is also the basic word order in Finnish, different clause types employ different word orders and the word order is function-based [20]. Whereas many SVO languages, including English, make use of prepositions, Finnish commonly makes use of postpositions. Such differences ought to be taken into account when comparing typologically distant languages, as they can affect methodological choices and data handling, and even level of analysis.

### Soundscapes and auditory experiences

The act of verbally describing auditory experiences is of interest to several fields of research, including soundscape studies, cognitive and sensory linguistics, as well as intermodal/-semiotic translation studies (in terms of accessibility). Soundscape studies especially [12,14,26] has shaped into an interdisciplinary research area with a focus on the meaningfulness of sound across the human experience, including sound sensations, listening practices and acoustic design [27, p. 12]. Below, we focus on linguistically orientated approaches.

At its core, the human experience takes place through sensing: seeing, feeling, hearing, tasting and smelling, often in a multisensory fashion [11,28]. These senses are coded into language (e.g., different color words), allowing individuals to share their experiences with others. Although more nuanced conceptualizations exist [27], for the purposes of this article we use “sensory experiences” to refer to the combined act of perception and interpretation which an individual pursues when making sense of the world. Interestingly, however, different languages have (somewhat) different resources for describing sensory experiences [11, p. 51]. For example, there is variation in the number of color categories [29,30], how motion is expressed (e.g., satellite-framing in Germanic languages and verb-framing in Romance languages [31]), and how spatial references are expressed (egocentric vs. geocentric directions [32, pp. 114–116], [33]) in different languages. In addition, there are also individual differences in how people sense the world around them [11, pp. 48–50; 34], for example, based on visual abilities (see below). As such, exploring how the different senses are encoded in language has become a prominent field of study within cognitive linguistics, appropriately named as “sensory linguistics” [11].

Particular attention has been paid to lexical richness and variation, and, for example, classification and rating of sensory words [29,35–40]. For one, a “visual dominance” has been observed in English and many other languages, meaning that there is a much richer lexicon available for describing visual experiences compared to the other senses [9–11, pp. 55–58], see also [41]. It may simply be more difficult to describe some sensory modalities and experiences than others, although it is unclear whether this is “due to cognitive architectural constraints or due to the limits of language” [41, p. 421]. Considering the dominance of vision, it is especially interesting to consider how visually disabled individuals make sense of the world.

Although visual experiences are often considered the easiest to describe, auditory experiences seem to be easier to describe than tastes and smells [41, p. 415]. This is reflected in the lexicon, as there are relatively many lexical items that primarily describe auditory experiences, including common adjectives (*loud, quiet, audible, silent*) and verbs (*chirp, squeak, boom, hiss, clatter*), many of which are iconic or onomatopoetic [11, p. 151].

Previous studies on lexical variation and individual linguistic features have received some criticism. First, with an underlying assumption of words having stable and context-independent meanings, (comparative) vocabulary studies often fail to consider other ways of expressing similar meanings [13,42]. Second, previous studies have suffered from a western-centered bias, often specifically using English as the standard for comparisons [42, p. 354]. As such, more nuanced, multi-method analyses of linguistic diversity in sensory descriptions are needed [13], see also [5].

Moreover, perception studies have shifted from studying individual sounds to using more complex stimuli, including different types of soundscapes [13,43]. In natural environments, listeners regularly make perceptual judgments of a sound based on several acoustical features appearing in the soundscape [44]. What is essential in understanding soundscapes is that they are not merely perceived neutrally by individuals being exposed to them, but instead, they are experienced and interpreted subjectively. For example, different sounds can either annoy or please different individuals [12,29]. According to Hartman and Paradis [13, p. 450], “[a] soundscape thus encompasses an event that generates sound and an acoustic signal that is actively perceived by a listener vis-à-vis an experiential context, previous experiences, and current goals.” Moreover, an important part of the act of describing soundscapes is the ability to categorize the different sounds [26]. As our analysis also demonstrates, different people can interpret the same soundscape differently [13, cf. 11, pp. 48–50]: they make different choices as to which aspects of the experience they highlight — and which to describe in the first place [11, p. 52]. Much like Hartman and Paradis [11], we add to the field of linguistically orientated investigation of soundscape descriptions.

### Linguistic studies on descriptions of sensory experiences

Despite the fact that human-generated descriptions play an important role in training machine learning models and quality issues with training datasets have been acknowledged [7], aspects such as study design and biases in the datasets have received more attention (see [3,45]) than linguistic properties. The linguistic studies on training datasets have explored the difference between captions and descriptions [15], image-text coherence relations [46], negation [16], and human references [47], for example. Concerning the latter, van Miltenburg, Elliott and Vossen [47] illustrated that descriptions include highly subjective interpretations related to such aspects as ethnicity and attractiveness, for example [47, pp. 416–417].

Although we also focus on training datasets, our analysis more closely resembles that of Hartman and Paradis’ [13], situated in the field of cognitive linguistics. Hartman and Paradis provide a detailed, discourse-oriented analysis of 3 873 sound descriptions collected from 214 crowd workers, each participant describing 20 audio clips of everyday (human and non-human) sounds [13, pp. 453–456]. Identifying different event meanings (e.g., agents, interactions, and settings), sound meanings (quality [e.g., clunk, pop], properties [e.g., *high-pitched*], temporal structure [e.g., *continuous*] and spatial structure [e.g., *foreground*], as well as experience meanings (e.g., pleasantness, familiarity) in the descriptions, the authors showed that nearly all responses included a description of an event, whereas personal experiences were less commonly described [13, pp. 458–461].

Furthermore, while it is well-known that different languages offer different means to describe sensory experiences and that cultural factors play a role in aspect such as which features are considered salient enough to describe [17,48], systematic cross-linguistic studies on descriptions have been scarce, cf. [49]. One exception is a study by van Miltenburg, Elliott and Vossen [17], in which the authors compared image descriptions in three Germanic languages (English, German and Dutch), showing that there are indeed important differences in the descriptions produced even by speakers of related languages, especially in terms of identification of features, ethnicity markers, subjective interpretations, and negations. We add to the field with a cross-linguistic study of two unrelated languages.

### Perception and language use of visually disabled individuals

As noted above, individuals often have differences in their perception. Since our study includes visually disabled participants, we shortly discuss visual disability and perception studies in relation to language use. First, although people who are blind or partially sighted are often thought to be disconnected from the visual realm, this is not always the case. For instance, many visually disabled individuals retain residual vision and, thus, some functional sight in certain conditions. Even if there is no residual vision, non-congenitally blind individuals can retain visual memories. Previous research on interaction between blind and sighted persons also indicates that blind individuals may use second-hand knowledge to make connections to the visual world [50,51]. Thus, visually disabled individuals can make use of visual information when describing their experiences.

Moreover, it seems that visually disabled individuals may have improved abilities in other senses. For example, previous studies have indicated that visually disabled children perform average to above-average in tests measuring spatial-hearing abilities [52]. In another study, Gougoux et al. [53] investigated the neural basis of sound localization skills in visually disabled and sighted participants, concluding that visual deprivation from an early age could give a person an advantage in using spectral cues when localizing sounds. From the perspective of language production, it also seems that blind children may have a higher phonetic accuracy in their speech than sighted children [54]. Some differences in language production of blind and sighted adults have also been reported. For example, Mamus et al. [55] found that when verbalizing spatial representations, blind participants were more likely than sighted participants to mention landmarks and path of motion, especially in relation to themselves. However, Gougoux et al. [53] point out individual differences in stimuli processing among visually disabled participants, indicating the existence of intra-group differences. We add to such studies by comparing descriptions produced by sighted and visually disabled Finnish speakers.

## Data and methods

### Corpora

The data is sourced from the open SiVi-CAFE dataset, comprising three parallel corpora compiled with similar principles and using the same stimuli [56]. The corpora were attained in the fall of 2023, and the data includes no identifying information of the participants. The corpora were primarily produced for research on machine perception but provide much of interest to linguistic enquiries as well. The descriptions were collected by presenting 10-second audio clips to participants, each clip being described in written form by five different participants. The audio clips were selected from the TAU Urban Acoustic Scenes 2019 Development dataset [57]. The audio clips selected for the description task represent two acoustic scenes with sufficient similarity: public squares and urban parks in various European cities. Importantly, the same audio clips were used for the three corpora. The descriptions were collected with webtools (English data with Amazon Mechanical Turk, Finnish data with a separate tool), which allowed the participants to work independently, although this also means that situational factors (e.g., sound quality, ergonomics) were not controlled.

Corpus 1 includes Finnish descriptions by visually disabled participants (FI_VDP; source dataset visually_impaired-FI-original [56]), Corpus 2 includes Finnish descriptions by sighted participants (FI_sighted; source dataset sighted-FI-original [56]), and Corpus 3 includes English descriptions by sighted people (EN_sighted; source dataset sighted-EN-no_bias-original [56]). Sighted participants had either normal or corrected vision, whereas out of the participants with a visual disability 14 reported being blind, 9 partially sighted, and two did not provide details. The Finnish corpora include descriptions produced by Finnish speakers. In contrast, we assume that some of the English data (Corpus 3) were produced by non-native English speakers of varying backgrounds (i.e., international students at Finnish universities).

From the perspective of cross-linguistic comparison, this uncertainty of the speaker status of the participants is unideal, but the choice of corpora was dictated by data availability. In general, there is rarely any guarantee that English training datasets would be produced by native speakers, since dataset compilers typically only require residency in an English-speaking country [3]. Quality issues with these datasets are also well recognized (ibid.). In particular, although the English descriptions are generally understandable and mostly follow standard language conventions, we identified several features indicating L2 language use in the English data, including unidiomatic expressions (e.g., *walking up*) and common L2 errors (e.g., incorrect use of articles). Despite these issues, we judged the quality of the data sufficient for the descriptive analyses we pursued. Nevertheless, we considered the nature of the data carefully during the analysis, discussing the limitations the data poses further in the Discussion and conclusions section.

Since the datasets were compiled for machine learning purposes, acquiring a comparable amount of data was the target. However, recruiting large amounts of visually disabled Finnish speakers with sufficient digital skills proved challenging. The visually disabled participants (25) were thus requested to describe a total of 180 audio clips each, and all completed the assignment (Table 1). The recruitment of sighted Finnish speakers was also somewhat challenging, resulting in 42 participants, who were assigned to describe 90 audio clips each. However, some participants did not complete all the clips assigned to them, and the total number of descriptions is considerably lower than in the other corpora, reflected in corpus size as well. Lastly, the EN_sighted corpus consists of descriptions produced by 89 participants. Such differences in parallel data can become a problem in multidisciplinary research; if the same data are studied in different disciplines, such as signal processing and linguistics, the team should ideally plan the data collection together.

**Table 1:** Overview of corpora.

	Corpus 1 FI_VDP	Corpus 2 FI_sighted	Corpus 3 EN_sighted
Participant background	blind and partially sighted Finnish adults	sighted Finnish students	sighted non-Finnish students
Number of participants	25	42	89
Number of descriptions	4500	3612	4384
Number of words (total)	37 083	27 168	66 143
min. words per description	2	2	2
max. words per description	38	26	29
mean words per description	8.3	6.3	9.7
median words per description	7	6	9

The task instructions recommended working in short intervals, in a quiet environment with quality headphones. The participants were prompted to describe the audio clips with a one-sentence description, imagining they were describing the clip to a deaf person. They were further guided to describe all essential information about the situation as well as noteworthy details, with the exception that speech should not be transcribed, nor should one’s own reflections on the interpretation process. The participants were also informed that many audio clips might sound similar. Prior to the task, the participants were shown three example audio clips with example descriptions. While it was not controlled, some of the visually disabled participants likely used screen readers and speech-to-text applications. Despite some differences in the data collection (e.g., number of participants), we assess that the three corpora have been compiled consistently enough to allow for comparisons, taking into account data quality when discussing the results.

To allow for corpus-linguistic analyses, each corpus was POS-tagged *(Parts of Speech tagging*)*.* The Finnish corpora were automatically lemmatized and tagged with the software Mylly [58], which tags each item’s word class, and various levels of inflection (e.g., number, case, tense). However, the accuracy of the tagger was not satisfactory due to two main reasons: polyfunctionality, and more importantly, numerous spelling mistakes in the data, which appeared particularly in the VDP corpus. In the VDP corpus, these mistakes may partly be due to technical issues with the screen reader and/or text processor. The English data was POS-tagged and lemmatized with TagAnt [59], but due to an abundance of non-standard language use, L2-type errors and spelling mistakes, tagging accuracy was unsatisfactory. Hence, manual correction of the tags and lemmas was required with all three corpora. We corrected tags and spelling of the lemmas, running various error-checking procedures. Most errors were found in the adposition and adverb tags in the Finnish data, and in verb and noun tags with the English data. Importantly, we only corrected clear typing errors, but not nonstandard forms (e.g., *vas.* as short-hand for *vasemmalla* ‘on the left’). We also did not pursue correcting misspelt compound words, which are common (and commonly misspelt) in Finnish. Despite considerable efforts, some errors undoubtedly remain in the data. All three corpora were handled and analyzed in Microsoft Excel to support multiple annotators and both languages, with the exception of producing n-grams with AntConc [60].

### Methods

We analyzed the data at two different levels. First, we gained an overview of the three corpora with a descriptive corpus analysis, relying on frequency and n-gram analyses. Second, we selected a smaller sample of descriptions for a more detailed qualitative analysis. To reduce the number of language-external factors, we selected only audio clips recorded in parks. The small sample includes descriptions for 10 audio clips, each clip including 5 descriptions for each of the three parallel corpora, for a total of 150 descriptions.

**Fig 2:**
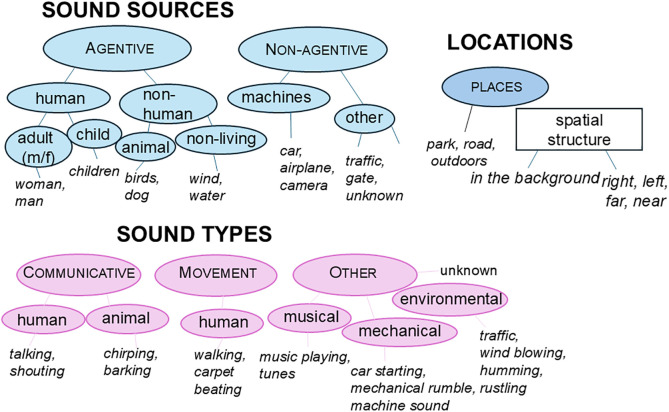
Content analysis coding scheme. Bolded capitalized labels reflect the upper level of the hierarchy, circled small capital labels represent middle-level; examples of specific codes are italicized. Boxed item separate from main coding scheme.

With the sample, we carried out a qualitative content analysis [61,62]. Although some frameworks for everyday sounds exist (see overview in [26]), we developed a data-driven coding framework during a pilot [62, p. 442], which included 50% of the data. While we explored different levels of coding during the pilot (e.g., linguistic variation), with the aim of examining which elements the participants had chosen to verbalize, we proceeded to simply code the content: 1. sound sources (e.g., *bird*), 2. sound types (e.g., *chirping*) and 3. locations (e.g*., park*, Fig 2). All researchers participated in the conceptual creation of the coding scheme in close collaboration, especially in terms of the upper and middle level categories (Fig 2). To ensure reliability and consistency of coding, check-up rounds were pursued by researchers who were not in charge of the original coding for sets of quasi-randomly selected cases. Since the coding itself was relatively straightforward (remaining mostly on the level of content, e.g., *bird,* Fig 2), there were only a few inconsistencies that were solved in mutual agreement.

As sound sources we coded both agentive actors (human and non-human, living and non-living), and non-agentive sources (Fig 2). Notably, wind and water (incl. *waterfall, stream, rain*) were considered agentive actors due to possessing a force of their own, the ability to instigate action [63, p. 16]. In principle, we coded sound sources as they appeared in the descriptions. For example, if a participant described the sound of high heels, we coded high heeled shoes as the sound source, even though the interpretation is likely that the sound is caused by a walking person. In other words, the coding closely followed the data. Sound types were divided into three subcategories: communicative sounds, sounds indicating movement by living creatures, and other sounds. Although rare, we coded named locations for the sounds as well (e.g., park). As an additional factor, we also coded descriptions of sound location itself (spatial structure).

Due to the amount and nature of the data (e.g., variation in naming practices), we mostly coded categories at the hypernym level, e.g., *bird* for different species of birds. However, we also counted the instances when the participants used detailed descriptions (e.g., *crow*) or described the sound types with descriptive attributes (e.g., *loud*, see [13, p. 458].

## Results and analysis

### Overview of corpora

To gain an overview of the data, we conducted different types of frequency analyses of the corpora. Since we were interested in lexical richness, we counted the number of lemma types at the word class level, calculating normalized frequencies to allow for comparison between the corpora (Table 2). As Table 2 illustrates, the FI_sighted corpus (Corpus 2) shows the highest frequencies for most of the categories, however the greatest difference seems to be between Finnish and English corpora, as there is much less variation in the EN_sighted corpus, particularly in the use of nouns, verbs, adjectives and adverbs (Table 2).

**Table 2:** Normalized frequencies (per 10 000) for the number of lemmas in each word class.

	Corpus 1 FI_VDP	Corpus 2 FI_sighted	Corpus 3 EN_sighted
Nouns	331	372	137
Verbs	136	178	64
Adjectives	90	107	45
Adverbs	57	65	23
Adpositions	11	11	7
Pronouns	7	9	5
Particles	9	12	3
Numerals	2	4	2
corpus size (absolute freq.)	37083	27168	66143

To summarize lexical richness, we calculated the type-token-ratio (TTR) for a sample of 20 000 tokens in each corpus (Table 3). We worked with lemmatized data since Finnish, as an agglutinative language, includes more inflectional variation than English, and since it has been suggested that L2 speakers may not make use of morphological complexity to the same extent as native speakers [64]. In other words, un-lemmatized data produced by presumably native Finnish speakers could appear richer than the English data including at least some L2 usage. Nevertheless, despite working with lemmatized data, the results demonstrate greater variation among the Finnish corpora (TTR 0.08 and 0.09), compared to the English corpus (0.05) (Table 3). It is also to be noted that the use of lemmatized data deflates the TTRs, however, even when considering unlemmatized data, the TTR for each of the complete Finnish corpora is 0.14 and 0.04 for the English corpus. Another factor deflating lexical diversity is the nature of the data: the individual descriptions are short [64] and the participants reacted to the same stimuli, hence often using similar vocabulary (see [8]).

**Table 3:** Type-token-ratio for lemmas.

	Corpus 1 FI_VDP	Corpus 2 FI_sighted	Corpus 3 EN_sighted
lemma types	1664	1776	984
tokens	20 000	20 000	20 000
TTR	0.08	0.09	0.05

To gain an overview of the aspects the participants had verbalized, we also investigated bigrams, which are less frequent overall in the Finnish corpus due to structural differences between the two languages. Nevertheless, the bigrams across the three corpora illustrate that sounds from animate sources (‘*people’, ‘birds’*) are described frequently, as are sounds related to traffic (we use single quotes in combination with italics to indicate that the item is both a linguistic example and a translation, i.e., it was employed in both languages). What is also apparent is that the participants have often identified more than one sound source or type, as they are using the conjunction ‘*and’* frequently. The bigrams further illustrate some common linguistic realizations, supported by our qualitative analysis and closer reading of the data (presented below). For one, the Finnish descriptions seem to include frequent use of nominal phrases (e.g., *liikenteen melua,* ‘noise of traffic’), although verbal phrases also appear (e.g., *joku kävelee,* ‘someone is walking’) (Table 4, translations provided in Table 7 in S1 Appendix). In contrast, the English descriptions seem to make use of different types of -ING phrases instead, often without the presence of a finite verb (e.g., *people talking, birds chirping*).

**Table 4:** Top 15 bigrams, absolute frequencies of unlemmatized data.

Rank	Corpus 1 FI_VDP	freq.	Corpus 2 FI_sighted	freq.	Corpus 3 EN_sighted	freq.
1	liikenteen melua	262	puhetta ja	178	in the	2004
2	ihmisten puhetta	260	joku kävelee	164	the background	1462
3	puhetta ja	255	ihmisten puhetta	157	people are	832
4	liikenteen kohinaa	155	linnut laulavat	143	people talking	799
5	joku kävelee	147	taustalla kuuluu	126	and a	722
6	lintujen laulua	147	ihmiset puhuvat	124	are talking	708
7	ja liikenteen	144	lintujen laulua	121	birds chirping	621
8	ääniä ja	144	ja taustalla	117	a person	594
9	liikenteen ääniä	130	ääniä ja	107	talking and	560
10	ihmisten ääniä	128	liikenteen ääniä	97	birds are	507
11	ja ihmisten	126	mies puhuu	86	talking in	472
12	ja taustalla	120	ja joku	82	the end	456
13	linnut laulavat	119	nainen puhuu	78	far away	450
14	lasten ääniä	110	ja lopussa	75	at the	428
15	taustalla liikenteen	107	lintu laulaa	75	sound of	416

We also note that in terms of spatial structure, sounds in the English corpus are often described to appear in the *background,* or infrequently *in the back,* but rarely in the *foreground* (Table 5). The same trend appears in the Finnish corpora, although the VDP corpus includes more instances of *etualalla* (‘in the foreground’) and the sighted corpus more instances of *tausta-[NOUN]/taustalla* (compound nouns with ‘background’, and ‘in the background’), included were also much more infrequent instances of *edessä* (‘in the front’), *edustalla* (‘in the foreground’), and *takana* (‘in the back’), *taka-alalla* (‘in the background’). However, the VDP corpus includes much more variation, including combinations with egocentric directions, e.g., *etuoikealla* (‘in the right foreground’) and *etuvasemmalta* (‘from the left foreground’).

**Table 5:** Normalized frequencies (per 10 000 words) for notions of spatial structure.

	Corpus 1	Corpus 2	Corpus 3
variants of...	FI_VDP	FI_sighted	EN_sighted
background	264	309	254
foreground	16	6	2
left	119	4	0
right	107	3	0
corpus total	37083	27168	66143

Notably, variations of ‘left’ and ‘right’ appeared much more frequently in the VDP corpus than in the other two corpora, again with a lot of variation: *vasen* and the short-hand *vas* ‘left’*, vasemmalle* ‘to the left’, *vasemmalla* ‘on the left’, *vasemmalta* ‘from the left’*, vasemmalle* (‘to the left’), *etuvasemmalla, etuvasemmalta* (*etu*- for ‘frontal’), *takavasemmalla* (*taka*- for ‘posterior’), *vasenvoittoinen* (‘more-to-the-left’)*.* With similar variations, ‘right’ appears almost as frequently as ‘left’. Curiously, *left* and *right* do not appear in the English corpus at all (Table 5). It is possible this is due to language-external reasons, such as sound quality, since a different file format was used for the English data collection.

### What happens in parks?

In this section, our attention focuses on the sample of 150 descriptions. While the analysis is qualitative in nature, we counted the occurrence of different elements the participants had identified and verbalized. In addition, we counted the number of sound events, to use the term loosely, to refer to the verbalizations of the different sound events the participants chose to describe. For example, example 1 includes descriptions of two sound events (*music, birds chirping*), and example 2 includes three events (*talking, laughing and bird chirping*).

(1) *Music with birds chirping in background.* (EN_sighted, clip 4)

(2) *People talking and laughing while faint bird chirping can be heard in the distance.* (EN_sighted, clip 6)

Overall, the three groups described a somewhat different number of sound events; the visually disabled group has the highest total number of sound events (Table 6). Moreover, in none of the audio clips did all participants from the three groups (n = 15) describe the same number of sound events. The VDP sample has the highest maximum number of words overall, and the number of words and words per sound events is considerably higher than for the sighted Finnish sample. As an analytic language, the word count for English is inflated by articles and prepositions, but as will be demonstrated below, the language use in the VDP sample seems to be the richest qualitatively.

**Table 6:** Number of words and sound events in the sample of 150 descriptions, absolute frequencies.

		Sample 1 FI_VDP	Sample 2 FI_sighted	Sample 3 EN_sighted
Number of words	Min.	3	5	4
	Max.	29	19	21
	Median	7	6	10
	Total	472	354	495
Sound events	Min.	1	1	1
	Max.	5	5	4
	Median	2.5	2	2
	Total	132	126	110
Number of words per sound event	Min.	1.7	1.7	2.5
	Max.	9.7	6.0	10.5
	Median	3.3	2.5	4.6

**Fig 3:**
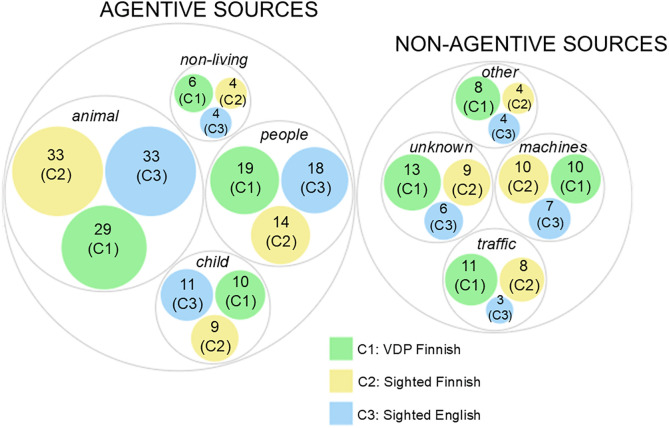
Circle-packing chart of sound sources, absolute frequencies.

**Fig 4:**
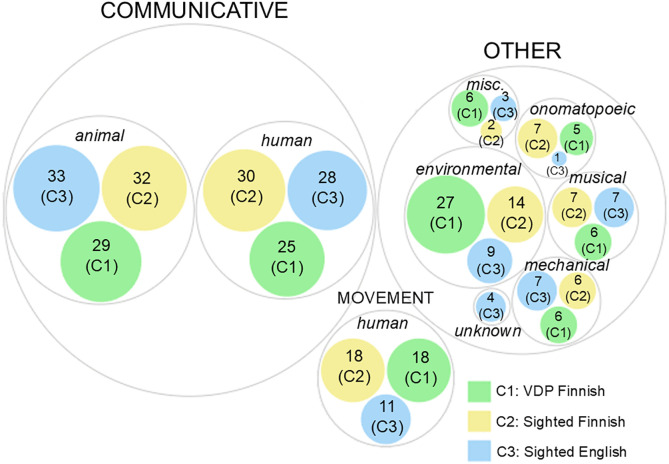
Circle-packing chart of sound types, absolute frequencies.

The content analysis below demonstrates that the three groups described similar aspects overall, although there was variation within audio clips. Our hierarchical coding scheme included three main categories: sound sources, sound types, and locations (see Fig 2). The results for the first two are presented as circle-packing charts (Figs 3 and 4), created with RawGraphs [65]. In each chart, the size of the circle is relative to the frequency of the element within the dataset, the three corpora marked by different colours (corpus number in parentheses).

The most common sound sources identified in the parks were those of humans and birds. With humans, the participants most often described these actors as ‘*people’*, and only in about a dozen cases the gender of the actors was specified as either female or male. It may be that the participants used gender to distinguish between actors (e.g., example 16 below), or that in specific audio clips, gender was a particularly salient feature, in contrast to potentially more muffled sounds of people as a collective. When considering the whole corpora, there are several hundred mentions of gender. Similarly, at times the participants specified ‘*children’* as the sound source. When doing so, some participants designated other human talk to ‘*adults’*, perhaps to avoid indicating children as non-people, while others did not (‘people’, ‘child’*,* 3). In contrast to adding details, unspecific human references with *someone* also occurred a few times in the sample (see example 7). For each example, we provide approximate translations after the glossed examples; the extracts are translated to resemble the original ones as closely as possible (e.g., word order might be unidiomatic to English).

Abbreviations in the interlinear glosses follow the Leipzig Glossing Rules (https://www.eva.mpg.de/lingua/resources/glossing-rules.php), complemented with ade = adessive, ess = essive, ill = illative, ine = inessive, par = partitive, trasnl = translative.

(3) *Ihmise-t* *juttele-vat* *ja* *lapsi* *huuta-a* *iloisesti,*

people-pl chat-3pl and child shout-3sg happily

*kuulu-u* *linnu-n* *laulu* *ja* *kaukaisuude-ssa*

be.audible-3sg bird-gen song and distance-ine

*lentä-ä* *lentokone.*

fly-3sg airplane

‘People chat and a child shouts happily, a bird’s song is audible and in the distance an airplane flies.’ (FI_VDP, clip 3)

The sounds allotted to humans were of two types: communicative sounds and movement sounds (Fig 4). Communicative sounds mostly comprise descriptions of ‘*speech*’ (e.g., *talking*, *chatter, freq.* 48), but descriptions of ‘*laughter*’ (*freq.* 12) and ‘*shouting*’ (or ‘*screaming*’; *freq.* 22) appeared as well, the latter often assigned to children (4). There was also a singular description of a woman singing. Sometimes, the sound descriptions were vague, e.g., ‘*human voices*’.

(4) *Birds singing some close, and some far, a child shouting and telling something loudly*. (EN_sighted, clip 3)

Movement sounds mostly include references to ‘*walking*’ (*freq.* 32) either as such or by describing the sound of footsteps, more common in the Finnish data. Interestingly, sometimes the end of the movement sound was also described (5). With movement sounds, both Finnish groups showed more variation than the English group; the visually disabled group turned out to be quite expressive, describing even very specific acts (examples 6–7). In a few cases, sounds related to breathing, spitting and smacking of the lips were described, considered movement sounds.

(5) *Etää-ltä* → *kuulu-u* → *huuto.* → *Kaue-mpa-a*

far-abl → be.audible-3sg → shout → Far-cmpr-par

*oikea-lta* → *kuulu-u* → *liikentee-n* → *kohina.*

right-abl → be.audible-3sg → traffic-gen → hum

*Lähe-mpä-nä → oikea-lla → kolme → nopea-ta*

close-cmpr-ess → right-all → three → quick-par

*askel-ta, → jo-i-sta → viimeinen → on → pysähdykse-n → ääni*.

step-par → which-pl-ela → last → be.3sg → halt-gen → sound

‘From afar a shout is audible. From further on the right the hum of traffic is audible. From closer on the right three quick steps, of which the last is the sound of halting.’ (FI_VDP, clip 10)

(6) *Linnu-n* → *piipitys-tä* → *ja* → *lehde-n* → *sivu-j-en* → *kääntely-ä.*

bird-gen → beeping-par → and → magazine-gen → page-pl-gen → turning-par

‘Peeping of a bird and turning of magazine pages.‘ (FI_VDP, clip 1)

(7) *Joku → kävele-e → ja → heittä-ä → lopu-ksi → jo < ta > kin*

someone → walk-3sg → and → throw-3sg → end-transl → something<par>

*roskakori-in,* → *tausta-lla* → *liikenne* → *kohise-e.*

garbage.bin-ill → background-all → traffic → hum-3sg

‘Someone walks and throws in the end something into the garbage bin, in the background traffic hums.’ (FI_VDP, clip 10)

Although not all movement sounds were explicitly assigned to human actors (*huudahdus,* ‘shout’ in 8), we interpreted them as human activities; it seems likely that the participants are processing and describing the audio clips from a humancentric perspective, thus leaving human actors unmarked. While sounds denoting animal movement might have been described, none appeared in the sample. When inspecting the complete corpora, there are a few dozen instances of bird movement (‘flying’, ‘wing’).

(8) *Kuulu-u* → *humina-a,* → *kaukainen* → *huudahdus* → *ja*

be.audible-3sg → hum-par → distant → shout → and

*askel-i-a,* → *jo<t>ka* → *päätty-vät* → *seisahdukse-en.*

step-pl-par → that-pl → end-3pl → halt-ill

‘Humming is audible, a distant shout and [foot]steps, which end in a halt.’ (FI_sighted, clip 10)

Although Figs 3 and 4 show sound source and sound frequencies for animals, in actuality, most animal sounds and sources are references to birds (*freq.* 92), and there were only a few mentions of dogs, and curiously, one visually disabled participant described a sound resembling the mooing of a cow (*lehmän ammunnan kaltaista ääntä*). Bird sounds were typically described as ‘*singing’*, or more descriptively as ‘*chirping’*; a few times unspecific descriptions such as *birds making noise* appeared as well*.* The species of the birds was typically not mentioned, with the exception of crows, whose identification seems to be due to the distinguished quality of their communicative sound, which was typically described as *cawing* (*raakkua),* or *crying.* In example 9, the sound is further described onomatopoetically as “kraa” in quasi-phonetic spelling.

(9) *Birds chirping and crows caw* *(”kraa” sound).* (EN_sighted, clip 5)

While most agentive actors are birds and humans, there were also 14 instances of non-living actors, including agentive actors: ‘*wind’* and references to water (10). Included as environmental sounds (Fig 4), the sound of the wind was described in English by the active verb *blowing* or as *wind noise*, whereas in Finnish, the active verb *humista* (‘to hum’) was typically employed; sometimes the deverbal nominalization *humina* (‘the hum’/’the humming’) was used instead.

(10) *Erilais-i-a → lintu-j-a → laula-a, → vesiputous → kohise-e*,

different-pl-par → bird-pl-par → sing-3sg → waterfall → rush-3sg

*koira* → *ulvo-o.*

dog → howl-3sg

‘Different [types of] birds sing, a waterfall rushes, a dog howls.’ (FI_sighted, clip 9)

Non-agentive sound sources were less frequent, including different types of machines (e.g., *car, radio, camera, chain saw; lawnmover* and *machine* in examples 11–12), traffic, unknown sources and miscellaneous sources such as *a branch*. Specific references to cars were included as machines, even though the sound of cars could also be conceptualized as traffic. Yet typically car-specific references conveyed the sound of individual cars (11), whereas traffic represents a collective of different types of vehicles.

(11) *Puisto-ssa* → *kuulu-u* → *liikente-en* → *melu-a* → *sekä*

park-ine → be.audible-3sg → traffic-gen → noise-par → and

*ruohonleikkuri-n* → *voimakas-ta* → *ään-tä.*

lawnmover-gen → strong-par → sound-par

‘In the park traffic noise is audible as well as the loud sound of a lawnmower’ (FI_sighted, clip 12)

(12) *A brief machine sound, far way machine moving leaking sound.* (EN_sighted, clip 10)

The sounds associated with non-agentive sources are collected under other sounds in Fig 4. Sounds of the city and traffic were included as environmental sounds*,* with water and wind sounds. In Finnish, the sound of traffic was typically described as noise (*liikenteen melu* ‘noise of the traffic’), rumble (*liikenteen pauhu* ‘rumble of the traffic’), or static noise (liikenteen kohina ‘hum of the traffic’), whereas in English it was only ever described as *traffic noise*; curiously, traffic was referenced only three times in the English sample, whereas the Finnish groups referenced it more frequently. Moreover, often explicitly connected to the environment or background (examples 13–14), the environmental sounds seem to be perceived as continuous, in contrast to shorter sounds which presumably have a beginning and an end in the audio clip (see “discrete interactions”, [66]). Indeed, shorter or solitary occurrences were often described with specific sound words, which convey the momentariness of the sound morphologically. In the Finnish data, this was typically achieved with nouns like *naksahdus* ‘click’, *narahdus* ‘creak’, *napsahdus* ‘snap’, *kumahdus* ‘boom’ and *poksahdus* ‘pop’, which are derived from verbs expressing momentariness with the derivative suffix *-(A)htA* and complemented with the suffix *-Us* used to derivate nouns from verbs [24]. Alongside other nominal sound words such as *rapina* ‘rustling’, and *kolina* ‘clunk’, these were considered onomatopoeic. *Kohina* ‘static noise’ and *humina,* or *humming,* can also be considered onomatopoeic; however, these sound descriptions were frequently explicitly associated with the city, traffic, wind or water, hence, they were included in environmental sounds. Certainly, also some of the descriptions of bird sounds were onomatopoeic, but they were included under a more salient category, i.e., communicative sounds.

(13) *A child shouting in a humming environment.* (EN_sighted, clip 7)

(14) *Kaupungi-n* → *kohina-n* → *seasta* → *erottu-u*

city-gen → noise-gen → admist → stand.out-3sg

*vaimea* → *matala-n* → *moottori-n* → *ääni* […].

faint → low-gen → engine-gen → sound

‘From amidst the noise of the city stands out the faint sound of a low engine.’ (FI_VDP, clip 4)

Musical sound types typically include references to music in general, or sound descriptions of specific instruments (15). Under other sound types we also included both unknown sounds and a handful of miscellaneous sounds from each group. The miscellaneous sounds include, e.g., *interference sound, snapping a branch,* and explicit references to clattering sounds made by shoes (16).

(15) *Ajoneuvo-j-en* → *äänt-ä* → *ja* → *jousisoittime-lla*

vehicle-pl-gen → sound-par → and → string.instrument-ade

*tuote-ttu-j-a* → *sävelm-i-ä* → *tausta-lla*.

produce-pcpl-pl-par → melody-pl-par → background-ade

‘The sound of vehicles and melodies produced by a string instrument in the background.’ (FI_sighted, clip 12)

(16) *Kengä-t* → *kopise-vat,* → *mies* → *puhu-u*

shoe-pl → clatter-3pl → man → talk-3sg

*ja* → *nainen* → *naura-a.*

and → woman → laugh-3sg

‘Shoes clattering, a man talks and a woman laughs.’ (FI_sighted, clip 7)

Explicit descriptions of unknown sound types (4 instances) curiously appeared only in the English data (17). In these cases, the participants have not named the source nor the sound, conveying uncertainty and/or difficulty in describing the sound. Unknown sound sources in contrast appeared among all three groups (Fig 3); particularly with sound descriptions such as *humming* or *rustling,* the sound source was simply left unnamed (18). We also note that some of the humming comments may relate to audio interference. In a similar fashion, sometimes the source was unspecific although the sound was described (19).

(17) *Unrecognizable sound in the background, a bird singing three times.* (EN_sighted, clip 9)

(18) *Yleis-tä* → *humina-a* → *pari* → *kerta-a*

General-par → humming-par → a.few → time-par

*varis* → *vaakku-u.*

crow → caw-3sg

‘General humming a crow caws a few times.’ (FI_VDP, clip 5)

(19) *Linnu-t* → *laula-vat* → *ja* → *jokin* → *kolise-e.*

Bird-pl → sing-3pl → and → something → rattle-3sg

‘Birds singing and something rattling.’ (FI_sighted, clip 1)

When it comes to describing other sounds, the visually disabled Finnish speakers showed the most variation (*freq.* 50), compared to both sighted Finnish (*freq.* 36) and English (*freq.* 27) participants. The one participant in the sample to describe silence was also visually disabled (20); at least in this sense, it seems like visually disabled people have somewhat richer and subtly different resources for describing sounds.

(20) *Melko* → *hiljainen* → *ympäristö,* → *jossa* → *tausta-lla*

quite → quiet → environment → where → background-ade

*kuulu-u* → *lintu-j-en* → *ään-tä,* → *sekä*

be.audible-3sg → bird-pl-gen → sound-par → and

*erittäin* → *kaukaa* → *kuulu-u* → *ihmise-n*

very → far → be.audible-3sg → human-gen

*puhe-tta*.

talk-par

‘A fairly quiet environment, where in the background the sound of birds is audible, as well as from a very far distance the sound of people talking is audible.’ (FI_VDP, clip 5)

Against expectations, the participants rarely referenced imagined locations for the sounds, even though the instructions specified in which type of places the audio clips had been recorded. Despite our flexible coding criteria including for example types of roads as locations (21), there were only 16 instances of locational references in our sample. Eight of these references were produced by the visually disabled group, five by the sighted Finnish group and only three by the English group. Cities, parks and outdoors were mentioned a few times, and one Finnish participant imagined the sounds to be heard from within an ice-skating rink.

(21) *Linnu-t* → *sirkutta-vat,* → *rollaattori-lla* → *aje-taan* → *hiekkatie-llä*,

bird-pl → chirp-3pl → walker-ade → drive-pass → gravel.road-ade

*matto-j-a* → *tampa-taan,* → *ja* → *lintu* → *visertä-ä*.

mat → beat-pass → and → bird → twitter-3sg

‘Birds are chirping, a walker is being driven on a gravel road, rugs are being beaten, and a bird is twittering.’ (FI_VDP, clip 1)

In addition, although the participants often described the different sound sources and sound types at a fairly general level [67, reprinted in 26, p. 187], we also counted the number of instances the participants described these aspects in greater specificity (e.g., *crow* instead of *bird, chirping* instead of *singing*). In this regard, the three samples faired similarly, as about 50% of the descriptions in all three samples included specific references. Following Hartman and Paradis [13, p. 458], we also counted the number of times the participants directly described sound types with different attributes (e.g., ‘*loud’, ‘quiet’, ‘fairly’,* example 20). In this regard, the VDP sample included the most attributes, a total of 38, whereas the sighted Finnish sample included 16 such instances, and the English sample only 12.

Last, we also paid attention to how the participants were verbalizing the location of the sounds in the audio clips (spatial structure). First, the order of the verbalizations likely indicated temporal occurrence, as is evident in example 21 where birds are described in the beginning of the description and a single bird in the end. Second, considering explicit sound descriptions, in the English sample 72% of the descriptions include notions of spatial structure, while in the sighted Finnish sample only 38% do, and in the VDP Finnish sample 56% do. Similar properties were described in both languages; the temporal location was described by indicating that the sound appeared ‘*in the end*’ or ‘*in the beginning*’ but not in the middle. Only in a few cases did participants describe temporal relationships with ‘*then*’ and ‘*while*’. Sounds were also perceived to differ in distance: ‘*in the background*’, ‘*in the distance*’, ‘*far away*’, or less often ‘*nearby*’, with variations in both languages. In addition to these, the visually disabled Finnish speakers described egocentric directions by indicating sounds to appear ‘on the right’ (*oikealla*) or ‘on the left’ (*vasemmalla*); the other two samples did not include egocentric directions, aligning with the results from the corpus analysis. In fact, with the exception of ‘*in the background*’, the VDP group used notions of spatial structure the most often also at the corpus level, as demonstrated in Table 5.

## Discussion and conclusions

We investigated soundscape descriptions produced in English and Finnish, the latter produced by both sighted and non-sighted participants. Overall, our analyses demonstrate that while there are many similarities, especially in terms of the aspects the participants have chosen to verbalize in their descriptions, there are also important differences. For one, the participants did not always describe the same actors or events, and the level of detail varied greatly, indicating that different people may make different interpretations of the soundscapes, and/or that different people make different choices as to what is salient enough to describe [13,67].

Previous research has indicated that when participants are reacting to the same soundscapes, free-text descriptions can still be lexically diverse, although overall TTRs tend to be low [8]. Indeed, the overall TTR for each of the corpora in our study were low (ranging from 0.05 to 0.09). Based on variation in the use of unique items in different word classes and the TTR, we found that the Finnish descriptions are more varied in terms of lexical diversity than the English descriptions, at the level of corpora. This difference seems striking, especially considering that the English descriptions were produced by the highest number of participants (89 in total), which should increase lexical diversity, not deflate it. Similarly, the English corpus had the highest average word count per description, but we attribute this to the morphological difference between English and Finnish. Considering these aspects, we ascribe the cross-linguistic difference at least partly to L2 usage present in the English dataset, a limitation we discuss in more detail below.

When comparing the two Finnish corpora, it turned out that although the visually disabled participants used more words on average, on the surface, the corpus produced by sighted participants seems to be somewhat richer in terms of lexical variation (i.e., slightly higher TTR and variation in word classes). In part this may reflect the fact that there were fewer visually disabled participants (25) than sighted Finnish participants (42). Indeed, the findings of our qualitative analysis more-so indicate that it was the language of visually disabled participants that was lexically richer and often more nuanced, e.g., in terms of described details and level of specificity. However, taking into account that there may be considerable inter-speaker variation (see Background), the nature of the data (relatively small number of visually disabled individuals producing the descriptions) restricts our ability to generalize the results.

During our content analysis, we developed a hierarchical taxonomy, categorizing the different aspects the participants described. Although the coding scheme was data-driven, it bears many similarities with a previous albeit broader categorization effort by Brown, Kang and Gjestland [35]. Our analysis of park soundscapes revealed that the participants most often described sounds by humans and birds. Previous research has also indicated that it is easier to distinguish between specific sound events than “collective background noise” [68, p. 55]. What is more, people seem to process sounds from living and non-living sources somewhat differently, relying more-so on semantic information with the former and more on acoustical properties with the latter [69], see also [26]. It thus seems that agentive sound sources are simply easier to identify, but it is also possible that sentient beings are considered more salient and thus verbalized more frequently than non-agentive sounds. Indeed, the human centricity was also evident in the way that some human-sourced sounds could be described without mentioning the source explicitly (e.g., ‘*a distant shout*’, ‘*footsteps*’, cf. [70]), whereas animal sounds seemed to require an explicit agent (e.g., ‘*birds chirping*’). Moreover, the type of actors (humans and birds) was typically not distinguished in more detail; this base level of specificity seems to be the preferred choice [26]. More detailed information would require more effort and might introduce uncertainty. This is likely why aspects such as gender or the species of birds were not commonly described by the participants, although a distinction based on age (*people* and *children*) was sometimes made. Like Hartman and Paradis [13, pp. 458–461] we also report that the participants in general did not commonly describe “experience meanings”, i.e., personal views on the sounds (e.g., *weird, annoying*), although in the Finnish data the participants often described the act of hearing (*‘kuuluu’*).

The qualitative analysis also demonstrated how the same sound could be described either based on the action which generates the sound (e.g., talking) or by the sound source (e.g., human sounds) [26]. Previous research has suggested participants to favour describing sound sources and events producing the sound [70] (see also [26]), which aligns with the results of the present study, as sound types (including actions such as talking) were described in over 90% of the sound events, and sound sources in about 70% of the sound events. Similarly, Hartman and Paradis [13, pp. 460–461] found that over 90% of their participants described sound events, collating what we call sound sources and sound types. We also noted that the participants described both discrete and continuous sounds [66]. Moreover, Hartman and Paradis [13] found that 64% of the participants described the quality of the sounds in more detail, referring to their properties (e.g., *loud*) or spatial structure (e.g., *background*). Although there was some variation between the participant groups of the present study, 40% to 70% of the time the participants described the spatial structure of the sounds, with the English sample including the most instances of sound locations. However, at the corpus level, the visually disabled participants clearly made the most use of egocentric directions (‘foreground’, ‘left’, ‘right’) with one exception (‘background’), which somewhat aligns with Mamus et al.’s [55] finding of blind participants describing paths of motion more commonly.

In the sample, we also noted that the VDP sample included more descriptive references to sound sources than the other two groups (e.g., ‘walker on a gravel road’, ‘rugs being beaten’). Indeed, we found that the visually disabled participants described sound properties the most frequently with attributes (38 instances), whereas the other two groups only had about a dozen such cases. However, we highlight that information about the properties of sound can also be imbedded in verbs (cf. *loud talking* vs. *yelling*). When considering all types of increased specificity (*bird* vs. *crow, talking* vs. *yelling* or *loud talking*), we found no considerable difference between the groups. While the results at different levels of analysis are somewhat contradictory, we suggest that, overall, visually disabled participants used somewhat more descriptive language, which may be due to being more accustomed to relying on their ability to recognize different sounds.

With the analysis of sentence-long soundscape descriptions in two different languages, we contribute to a new paradigm of sensory linguistics taking a discourse-oriented approach to account for the variety of linguistic resources in use when describing sensory experiences [13,42]. While our commentary on the structural differences between English and Finnish has been limited, we join Dubois et al. to call for more detailed cross-linguistic analyses taking into account the “diversity of linguistic devices”, both lexical and non-lexical [42]. For example, the [NOUN]+[-ING] seems to be a productive construction in English, and while infinitive constructions were sometimes used in Finnish too (e.g., [NOUN] + [-MA infinitive]), other type of constructions (e.g., nominalizations) seem to have been employed more frequently.

Indeed, one limitation of the study concerns the English dataset and the comparability of the Finnish and English corpora. At both the corpus and more detailed level of analysis, the English corpus turned out to include much less variation than the Finnish corpora. Although we hypothesize that this difference is at least partly due to the English corpus including L2 usage, we cannot conclusively demonstrate this without access to a parallel corpus by native English speakers. Thus, differences between the Finnish and English corpora cannot be attributed to cross-linguistic variation with certainty. Nevertheless, while this issue with data quality limited our ability to pursue a more nuanced structural comparison between Finnish and English, the issue itself highlights the importance of collecting examples of language use from speakers with sufficient skills in standard language, which could be assessed with qualification tests [7]. As such, when compiling training datasets, we advocate for employing participants with sufficient standard language skills. However, we emphasize this does not mean they must be native speakers of the language; some native speakers can lack skills in standard language use, while some non-native speakers may have excellent command of standard language norms [71,72, pp. 101–102].

In addition to exploring the variety of linguistic resources used in soundscape descriptions, these types of datasets provide avenues for examining the meanings that different linguistic structures used in soundscape verbalizations bring forth. Although falling beyond the scope of the present article, we want to highlight this type of meaning-construction studies as relevant areas for further inquiry. As Hartman and Paradis also suggest [13], future studies should investigate whether certain meaning components are verbalized as certain linguistic structures or constructions. As an example, manifestations of the [NOUN] + [-ING] in English (e.g., *birds chirping*) and the [NOUN] + [-MA] construction in Finnish (*lintuja laulamassa*) could be considered labelling events instead of directly describing the sounds themselves (cf. *linnun laulu,* ‘singing of the bird’). Thus, future studies could investigate in which ways different constructions emphasize different aspects of the soundscape (e.g., action, sound). Another question we were unable to explore in this paper concerns the foregrounding and backgrounding of information: are events presented linguistically in the same order as they are heard, or can they also appear according to some other criteria, such as salience? We might also ask what role different constructions play in the foregrounding and backgrounding of information. Last, we urge future studies to also consider these types of data from the perspective of grammatical norms. When lay people (of unknown language skills) are asked to describe different types of data points, the results might include many types of language use undesirable from a machine learning perspective.

## Supporting information

S1 Appendix Table 7. Approximate translations for top 15 bigrams (Table 4). (DOCX)
