# Supplementary material for: Linguistic diversity in machine learning training data: Content analysis of English and Finnish soundscape descriptions in audio captioning
Source: PLoS One. 2026 Jun 11;21(6):e0350043. doi: 10.1371/journal.pone.0350043 (PMC13257990; doi:10.1371/journal.pone.0350043)
Supplement: S1 Appendix — (DOCX) [file pone.0350043.s001.docx]

**S1 Appendix**.

*Table 7. Approximate translations for top 15 bigrams (Table 4)*

| Rank | Corpus 1  FI_VDP | freq. | Corpus 2  FI_sighted | freq. | Corpus 3  EN_sighted | freq. |
| --- | --- | --- | --- | --- | --- | --- |
| 1 | noise of traffic | 262 | talking and | 178 | in the | 2004 |
| 2 | people talking | 260 | someone walks | 164 | the background | 1462 |
| 3 | talking and | 255 | people talking | 157 | people are | 832 |
| 4 | rumble of traffic | 155 | birds are singing | 143 | people talking | 799 |
| 5 | someone walks | 147 | in the background [x] can be heard | 126 | and a | 722 |
| 6 | birds singing | 147 | people are talking | 124 | are talking | 708 |
| 7 | and traffic’s | 144 | singing of birds | 121 | birds chirping | 621 |
| 8 | sounds and | 144 | and in the background | 117 | a person | 594 |
| 9 | sounds of traffic | 130 | sounds and | 107 | talking and | 560 |
| 10 | sounds of people | 128 | sounds of traffic | 97 | birds are | 507 |
| 11 | and people’s | 126 | man talks | 86 | talking in | 472 |
| 12 | and in the background | 120 | and someone | 82 | the end | 456 |
| 13 | birds are singing | 119 | woman talks | 78 | far away | 450 |
| 14 | sounds of children | 110 | and in the end | 75 | at the | 428 |
| 15 | in the background traffic | 107 | bird sings | 75 | sound of | 416 |
